# Smear layer removal evaluation of different protocol of Bio Race file and XP- endo Finisher file in corporation with EDTA 17% and NaOCl

**DOI:** 10.4317/jced.54179

**Published:** 2017-11-01

**Authors:** Vahid Zand, Hadi Mokhtari, Mohammad-Frough Reyhani, Neda Nahavandizadeh, Shahram Azimi

**Affiliations:** 1 Assistant Professor, Department of Endodontics, Faculty of Dentistry, Tabriz University of Medical Sciences, Tabriz, Iran

## Abstract

**Background:**

The aim of the present study was to compare the amount of the smear layer remaining in prepared root canals with different protocols of Bio RaCe files and XP-endo Finisher file (XPF) in association with 17% EDTA and sodium hypochlorite solution.

**Material and Methods:**

A total of 68 extracted single-rooted teeth were randomly divided into 4 experimental groups (n=14) and two control groups (n=6). The root canals were prepared with Bio RaCe files (FKG Dentaire, Switzerland) using the crown-down technique based on manufacturer’s instructions and irrigated according to the following irrigation techniques: Group 1: XPF with 2 mL of 2.5% NaOCl for 1 minute. Group 2:, XPF with 1 mL of 17% EDTA for one minute. Group 3: XPF was used for 1 minute in association with normal saline solution. Group 4: XP-endo Finisher file for 30 seconds in association with 2.5% NaOCl and 17% EDTA for 30 seconds. The negative control group: NaOCl (2.5%) was used during root canal preparation, followed by irrigation with 17% EDTA at the end of root canal preparation. The positive control group: Normal saline solution was used for irrigation during root canal preparation. In all the groups, during preparation of the root canals with Bio RaCe file, 20 mL of 2.5% NaOCl was used for root canal irrigation and at the end of the procedural steps 20 mL of normal saline solution was used as a final irrigant. The samples were analyzed under SEM at ×1000‒2000 magnification and evaluated using Torabinejad scoring system. Data were analyzed with non-parametric Kruskal-Wallis test and post hoc Mann-Whitney U test, using SPSS. Statistical significant was defined at *P*<0.05.

**Results:**

The results of the study showed the least amount of the smear layer at coronal, middle and apical thirds of the root canals in groups 2, which was not significantly different from the negative control group (*P*<0.5).

**Conclusions:**

Under the limitations of the present study, use of a combination of NaOCl and EDTA in association with XPF exhibited the best efficacy for the removal of the smear layer.

** Key words:**Smear layer, XP-endo Finisher file, EDTA, Sodium hypochlorite.

## Introduction

Successful endodontic treatment involves the cleaning and shaping of the root canal system (1). Shaping of the root canal results in the formation of an amorphous layer, referred to as the smear layer, on the dentinal walls of the root canals (2,3). The presence of such a layer prevents the penetration of irrigation solutions and root canal obturation materials into the dentinal tubules, increasing the risk of bacterial infection and microleakage (4). In a systematic review and meta-analysis, Shahravan et al. concluded that removal of the smear layer improves the seal of the root canal system.

Several irrigation solutions have been used to decrease the remaining bacterial debris and necrotic tissues and the smear layer resulting from the preparation of the root canals (5,6). The most commonly used irrigation regimen for the removal of the smear layer consists of the use of sodium hypochlorite solution at different concentrations in association with 17% EDTA; in this regimen sodium hypochlorite solution serves as a tissue solvent and has an antibacterial role but it is very cytotoxic and if it is extruded from the apical foramen, it results in necrosis and periapical inflammation. It will be beneficial to replace sodium hypochlorite with other materials and techniques to remove the smear layer (7).

Considering the complexities of the root canal system, use of NiTi rotary instrument cannot result in proper debridement. Studies have shown that use of NiTi files for preparing the root canals only cleans 45-55% of the root canal walls. Concomitant use of NaOCl and EDTA, ultrasound or later yields better results to some extent (8).

Recently a NiTi rotary finishing file, referred to as XP-endo Finisher file (FKG Dentaire SA, La Chuux-de-tonds, Switzerland) has been introduced. This file has been recommended for use after instrumentation of the root canal to increase the efficacy of root canal cleaning with preservation of dentin. It has been reported that XP-endo Finisher can expand up to 6 mm in diameter when the file tip is squeezed, equal to 100-times of a corresponding-sized file. This results in the file’s ability to reach inaccessible areas (9,10).

XP-endo Finisher is a small file (ISO 25 in diameter and zero taper) with an increase in its flexibility.

Development and manufacture of XP-endo Finisher file relies on shape memory principle of NiTi alloy. This file is straight in phase M when it is cold but when it is exposed to the body temperature within the root canal, its shape changes because its molecular memory changes to phase A. Its shape in phase A in the rotational state allows access to and debridement of areas that the file has no access to in the standard state. The file returns to its straight shape when it becomes cold (11). The aim of the present study was to compare the amount of the smear layer remaining in prepared root canals with different protocols of Bio RaCe files and XP-endo Finisher file in association with 17% EDTA and sodium hypochlorite solution.

## Material and Methods

A total of 68 extracted single-rooted teeth with straight root canals, closed apices and no root canal calcification were randomly divided into 4 experimental groups (n=14) and two control groups (n=6).

The tooth crowns were removed to leave a root length of 15 mm. A #10 k-file was placed in the root canal so that it was visible under a magnification of X4 at the apical foramen. The working length was recorded at 1 mm short of this length.

The root canals were prepared with Bio RaCe files (FKG Dentaire, Switzerland) using the crown-down technique based on manufacturer’s instructions. The root canals were prepared up to file #35 with 0.4 taper in the apical area.

Group 1: During preparation of the root canals with Bio RaCe file, 20 mL of 2.5% sodium hypochlorite solution was used for root canal irrigation and at the end of root canal preparation, XP-endo Finisher file was used in association with 2 mL of 2.5% sodium hypochlorite solution for 1 minute.

Group 2: During root canal preparation, 20 mL of 2.5% sodium hypochlorite was used, and at the end of root canal preparation, XP-endo Finisher file was used in association with 1 mL of 17% EDTA for one minute.

Group 3: During the root canal preparation, 20 mL of 2.5% sodium hypochlorite solution was used, and at the end of root canal preparation, XP-endo Finisher file was used for 1 minute in association with normal saline solution.

Group 4: During the root canal preparation, 20 mL of 2.5% sodium hypochlorite solution was used, followed by the use of XP-endo Finisher file for 30 seconds in association with 2.5% sodium hypochlorite solution and 17% EDTA for 30 seconds.

The negative control group: Sodium hypochlorite solution (2.5%) was used during root canal preparation, followed by irrigation with 17% EDTA at the end of root canal preparation.

The positive control group: Normal saline solution was used for irrigation during root canal preparation.

In all the groups, at the end of the procedural steps 20 mL of normal saline solution was used as a final irrigant.

Evaluation under a scanning electron microscope (SEM)

The samples were analyzed under SEM at apical (3 mm from the apex), middle (7 mm from the apex) and coronal (11 mm from the apex) thirds at ×1000‒2000 magnification and photomicrographs were taken. The smear layer on the photomicrographs was evaluated using Torabinejad scoring system (11):

1. No smear layer: absence of any smear layer on the root surface, with open and clean dentinal tubules.

2. Moderate smear layer: absence of the smear layer on the surface, with dentinal tubules laden with the smear layer.

3. A large amount of the smear layer: complete coverage of the root canal walls with the smear layer, with the dentinal tubules laden with debris.

Data were analyzed with non-parametric Kruskal-Wallis test and post hoc Mann-Whitney U test, using SPSS. Statistical significant was defined at *P*<0.05.

## Results

**Figure 1:**
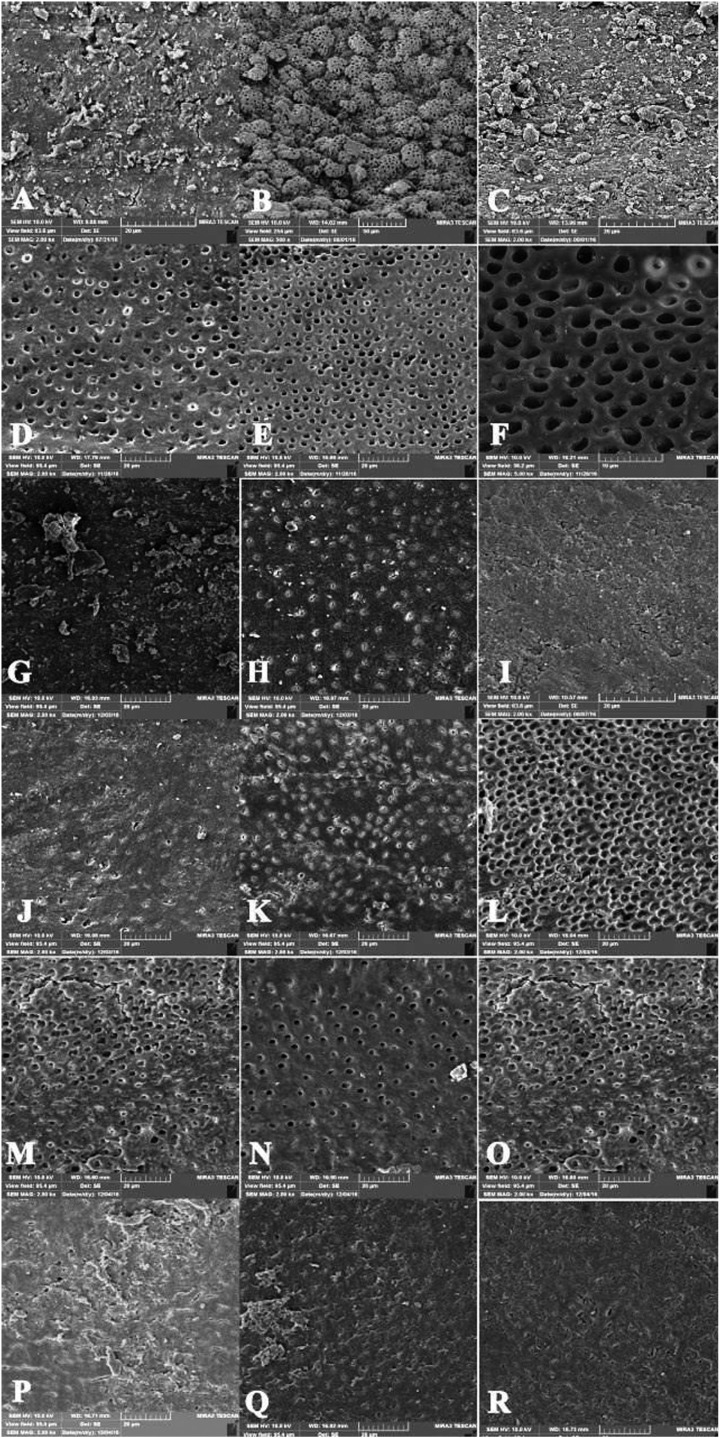
A) The apical third of the canal wall in group 1; absence of the smear layer on the surface, with dentinal tubules laden with the smear layer (score 2); B) The middle third of the canal wall in group 1 (score 2); C) The coronal third of the canal wall in group 1 (score 2); D) The coronal third of the canal wall in group 2; almost no smear layer is remained. Orifice of dentinal tubules are patent (score 1); E) The middle third of the canal wall in group 2 (score 1); F) The apical third of the canal wall in group 2 (score 1); G) The coronal third of the canal wall in group 3; complete coverage of the root canal walls with the smear layer, with the dentinal tubules laden with debris (score 3); H) The middle third of the canal wall in group 3 (score 3); I) The apical third of the canal wall in group 3 (score 3); J) The coronal third of the canal wall in group 4; (score 1); K) The middle third of the canal wall in group 4 (score 1); L) The apical third of the canal wall in group 4 (score 2); M) The coronal third of the canal wall in group 5; (score 1); N) The middle third of the canal wall in group 5 (score 1); O) The apical third of the canal wall in group 5 (score 1); P) The coronal third of the canal wall in group 6; (score 3); Q) The middle third of the canal wall in group 6 (score 3); R) The apical third of the canal wall in group 6 (score 3).

Root canal walls without any smear layer were detected in none of the study groups. Kruskal-Wallis test revealed statistically significant differences between the three root canal segments (coronal, middle and apical) and between all the irrigation solutions. The results of the study showed the least amount of the smear layer at coronal, middle and apical thirds of the root canals in groups 2 (Fig. 1), which was not significantly different from the negative control group (*P*<0.5).

**Figure 2:**
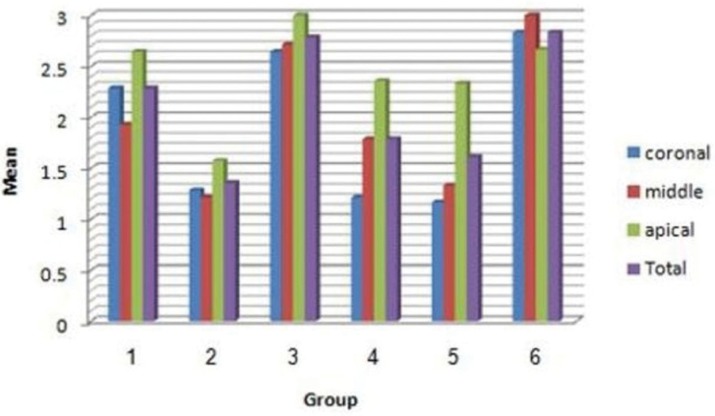
Mean of smear layer in different groups and different sections.

The amount of the smear layer remaining in the coronal third in group 4 was not significantly different from that in group 2, and the amount of the smear layer remaining in the middle and apical thirds was not significantly different from that in the negative control group (group 5). Irrespective of the cross-section, group 2 exhibited the least amount of residual smear layer, with no significant differences between group 4 and the negative control group (Fig. 2).

## Discussion

The smear layer is an irregular and amorphous layer that forms on the root canal walls after instrumentation (12). The unfavorable effect of the smear layer becomes manifested when microorganisms within this layer survive. In addition, the presence of the smear layer within the dentinal tubules might prevent penetration of sealer into the dentinal tubules, compromising the seal of the root canal filling materials (13,14).

Sodium hypochlorite solution is the most commonly used root canal irrigation solution (15). Alternative solutions are calcium-chelating agent such as EDTA. To increase the efficacy of the irrigation solutions, they should contact the root canal wall (16,17). Different techniques have been used with the use of syringes in order to remove bacteria, necrotic tissue debris and the smear layer. However, syringes can only transfer the irrigation solution up to 0‒1.1 mm beyond the needle tip, which is not sufficient for cleaning and debridement of inaccessible areas (18).

Recently a new instrument, referred to as XP-endo Finisher, has been introduced as a supplementary technique to improve the efficacy of root canal irrigation. This instrument undergoes expansion at body temperature; in addition, its helical movement within the root canal might result in its contact with the debris adhering to the root canal wall, removing them.

On the other hand, some studies have shown that a high rate of apical cleaning of the root canal has an important role in the elimination of bacteria from the root canal wall, demonstrating that the bacterial counts in the root canal decrease significantly with an increase in the apical size of the root canal. Bio RaCe rotary files (FKG Dentaire, La-chanx-de Fonds, Switzwerland) were introduced to increase the apical size. Based on the manufacturer’s claim, the chief aim of designing these files was to achieve a larger apical size for better debridement with less numerous files (9,10).

In a study by Khademi *et al.*, it was shown that the minimum apical cleaning of the root canal should be 0.30 mm (19). In the present study the minimum root canal cleaning was 0.04/#35.

In the present study, in general the results in groups 2 in relation to the removal of the smear layer with EDTA in association with XP at the end of root canal preparation was better than that in other groups, indicating that use of NaOCl alone is not sufficient and EDTA should be used along with it.

Alves *et al.* (20) showed a higher efficacy in removing the root canal obturation materials in curved root canals with the use of XP-endo Finisher file, which was confirmed in a study by Karamifar *et al.* (21), in which the use of XP-endo Finisher file resulted in the removal of more gutta-percha from the root canal walls compared to the control groups. The results of the present study are consistent with those of studies reporting that XP-endo Finisher file improves the efficacy of root canal debridement.

In the present study, in the coronal third of group 4 a combination of NaOCl and EDTA in association with XP-endo Finisher file was more effective in removing the smear layer, indicating that NaOCl is not properly carried to the middle and apical thirds. Therefore, the situation in group 2, in which only EDTA was used in association with XP-endo Finisher file, was better, which might be attributed to the deeper penetration of the solution and a greater diameter of dentinal tubules, resulting in greater effect of NaOCl in these areas. However, in the medical and apical areas an adequate amount of NaOCl does not penetrate into these areas and EDTA flows into these areas compared to NaOCl.

## Conclusions

Under the limitations of the present study, use of a combination of NaOCl and EDTA in association with XP-endo Finisher file exhibited the best efficacy for the removal of the smear layer.
